# Morphometric Evaluation of Changes in Alveolar Bone and Root Length During Mandibular Incisor Retraction in Class I Bimaxillary Protrusion: A Prospective Study

**DOI:** 10.7759/cureus.88414

**Published:** 2025-07-21

**Authors:** Vidhyawati Prajapati, Namit Nagar, Anupa Dave, Udisha Tripathi, Kaberi Lukhurakhon, Archita Bharadwaj

**Affiliations:** 1 Department of Orthodontics, Geetanjali Dental and Research Institute, Udaipur, IND

**Keywords:** alveolar bone, cone beam computed tomography, lower incisors, mandibular incisor retraction, retraction

## Abstract

Introduction

Orthodontic treatment often involves first premolar extraction and anterior tooth retraction to achieve aesthetic and functional harmony in bimaxillary protrusion cases. However, excessive retraction may cause complications such as root resorption and bone loss. This study aimed to evaluate morphometric changes in anterior mandibular alveolar bone and root length during retraction of the mandibular incisors in patients with class I bimaxillary protrusion using cone-beam computed tomography (CBCT). The objective of this study was to assess changes in alveolar bone width, height, and root length before and after retraction.

Materials and methods

This prospective observational study included 32 systemically healthy patients aged 18-30 years with class I bimaxillary protrusion requiring first premolar extractions. CBCT scans (pre- and post-retraction) were analyzed using standardized imaging parameters. Patients underwent orthodontic treatment with pre-adjusted edgewise appliances and temporary anchorage devices for maximum anchorage. The alveolar bone width, height, and root length were measured in the mandibular incisors. Intra- and inter-observer reliabilities were assessed, and the data were analyzed using paired t-tests and one-way analysis of variance (ANOVA), with a significance threshold of p < 0.05.

Results

Labial alveolar bone height, as determined by the vertical distance between the cementoenamel junction and alveolar crest, decreased significantly across all mandibular incisors post-retraction, whereas lingual bone height increased in all teeth, suggesting crestal bone apposition and resorption on the labial and lingual sides, respectively. The labial bone width decreased significantly, whereas the lingual bone width increased in all incisors, suggesting labial root movement with retraction. The root length was significantly reduced in all incisors, indicating the risk of root resorption. No significant intragroup differences were found among the incisors (p > 0.05); however, intergroup comparisons confirmed significant pre- and post-retraction changes (p < 0.05), indicating dynamic bone remodeling.

Conclusion

The retraction of the mandibular incisors in bimaxillary protrusion significantly altered alveolar bone dimensions and reduced root length with site-specific variations. CBCT proved essential for precise assessment, emphasizing the need for controlled retraction to balance aesthetic and functional outcomes while minimizing complications. Future research should explore long-term stability and strategies to mitigate root resorption.

## Introduction

Bimaxillary protrusion, a prevalent condition in the Asian population, is characterized by pronounced dentoalveolar flaring of the maxillary and mandibular anterior teeth, leading to lip protrusion and a convex facial profile that may be aesthetically displeasing [1]. In contrast to other populations, bimaxillary protrusion in Indians tends to be dentoalveolar rather than skeletal in origin, making it amenable to orthodontic correction without surgical intervention [2]. Class I bimaxillary protrusion often necessitates orthodontic intervention to achieve functional and aesthetic harmony [1]. The standard treatment protocol involves extraction of the first premolars, followed by retraction of the anterior teeth using maximum anchorage mechanics to reposition the incisors within the alveolar bone [1,3]. This approach aims to place the incisors upright within the medullary portion of the basal bone, optimize periodontal support, and balance the labial and lingual musculature for long-term stability [4]. Proper positioning enhances periodontal health and minimizes stress on supporting structures, contributing to favorable treatment outcomes [4].

However, excessive retraction of the anterior teeth can lead to iatrogenic complications, including root resorption, alveolar bone loss, fenestrations, dehiscence, and gingival recession [5]. These potential sequelae highlight the importance of understanding the therapeutic limitations of orthodontic tooth movement, particularly the concepts of "with-the-bone" and "through-the-bone" movements [6,7]. "With-the-bone" movement refers to repositioning teeth within the existing alveolar housing, while "through-the-bone" movement involves remodeling the bone, which may increase the risk of adverse effects [6,7]. An accurate assessment of these changes is critical for defining safe boundaries for orthodontic intervention.

Traditional imaging modalities such as lateral cephalograms, intraoral periapical radiographs (IOPA), and orthopantomograms have been widely used to evaluate orthodontic outcomes. The drawbacks associated with this methodology encompass obscured visual representations resulting from overlapping anatomical frameworks, as well as an absence of information pertaining to both the right and left aspects [8]. Goldberg et al. [9] indicated that periapical radiography is incapable of identifying resorption lesions measuring less than 0.3 mm in depth and 0.6 mm in diameter. The introduction of cone-beam computed tomography (CBCT) has revolutionized orthodontic diagnostics by providing three-dimensional (3D) imaging with high resolution and relatively low radiation exposure compared with conventional CT scans [10]. CBCT enables precise quantitative and qualitative evaluations of alveolar bone height, width, and root length, offering insights into changes that were previously difficult to assess using two-dimensional (2D) methods [10].

This study aimed to evaluate the morphometric changes in the alveolar bone during retraction of the mandibular incisors in patients with class I bimaxillary protrusion treated with first premolar extractions. The objective of this study was to assess the changes in alveolar bone width, alveolar bone height, and root length of mandibular incisors before and after retraction using CBCT.

## Materials and methods

Study design and setting

This prospective observational study was conducted at the Department of Orthodontics and Dentofacial Orthopedics, Geetanjali Dental and Research Institute, Udaipur, Rajasthan, India, from January 2024 to March 2025 with ethical approval from the Institutional Ethics Committee (Approval No. GU/HREC/2023/2206) in accordance with the Declaration of Helsinki. Written informed consent was obtained from all the patients.

Eligibility criteria

The inclusion and exclusion criteria were strictly defined to ensure a homogeneous sample and to minimize confounding variables. The inclusion criteria were as follows: systemically healthy patients aged 18-30 years diagnosed with dentoskeletal class I malocclusion with bimaxillary protrusion and crowding less than 2 mm in the mandibular anterior segment requiring extraction of the first premolars, bleeding and plaque index of 0 or 1, patients requiring retraction of mandibular anterior teeth > 4 mm, and presence of all permanent teeth except third molars. The exclusion criteria were as follows: root resorption prior to treatment, a history of previous orthodontic treatment, smoking, severe alveolar bone loss, abnormal root morphology such as dilaceration or short roots, presence of deciduous teeth, class II or class III malocclusions, a history of using antibiotics or corticosteroids in the past six months, pregnancy or lactation, craniofacial anomalies, bone disorders, and immunosuppression.

Sample size estimation

A minimum sample size of 32 patients was determined for the study, which was calculated using GPower software version 3.1.9.7 (Heinrich Heine University, Düsseldorf, Germany) based on the following parameters: 80% statistical power, 0.5% alpha error rate (two-tailed), and a two-independent-means repeated-measures t-test design. The effect size (d = 0.45) was derived from a prior study comparing pre- and post-retraction alveolar bone height changes in mandibular anterior teeth [11].

Methodology

A total of 64 CBCT scans (32 pre-retraction and 32 post-retraction) were collected from 32 patients. Demographic details were noted from all the patients. CBCT scans were obtained using the Carestream CS 9300 imaging system (Carestream Dental, Atlanta, GA, USA). The imaging parameters were standardized at an exposure of 90 kV, 6.3 mA, and 15 s, with a voxel size of 150 × 150 × 150 µm and a slice thickness of 1.1 mm. These settings ensured that the high-resolution images were suitable for detailed morphometric analysis. CS 3D Imaging software (Carestream Dental) was used for image processing and analysis.

All the patients underwent thorough oral prophylaxis one week prior to their bonding. Orthodontic treatment involved the extraction of the maxillary and mandibular first premolars to create space for anterior tooth retraction. The treatment was performed using a preadjusted edgewise appliance with a McLaughlin, Bennett, and Trevisi (MBT) prescription featuring 0.022 × 0.028-inch slot brackets (3M Unitek, Monrovia, CA, USA). The MBT system was chosen for standardized bracket prescriptions. Maximum anchorage was achieved using temporary anchorage devices (TADs), specifically OrthoEasy® miniscrews (Forestadent, Pforzheim, Germany), placed on the buccal shelf or interradicular areas to reinforce anchorage and minimize the mesial movement of the posterior teeth. The retraction phase involved en masse retraction of the anterior teeth using sliding mechanics with nickel-titanium closed-coil springs (G&H Orthodontics, Franklin, IN, USA) delivering a force of approximately 150 g per side in the mandibular arch. The treatment duration varied from 18 to 24 months depending on the individual patient response and the extent of retraction required. The mean retraction time was 6.34 ± 2.18 months. All patients were treated by the same orthodontist with more than 10 years of clinical experience.

**Figure 1:**
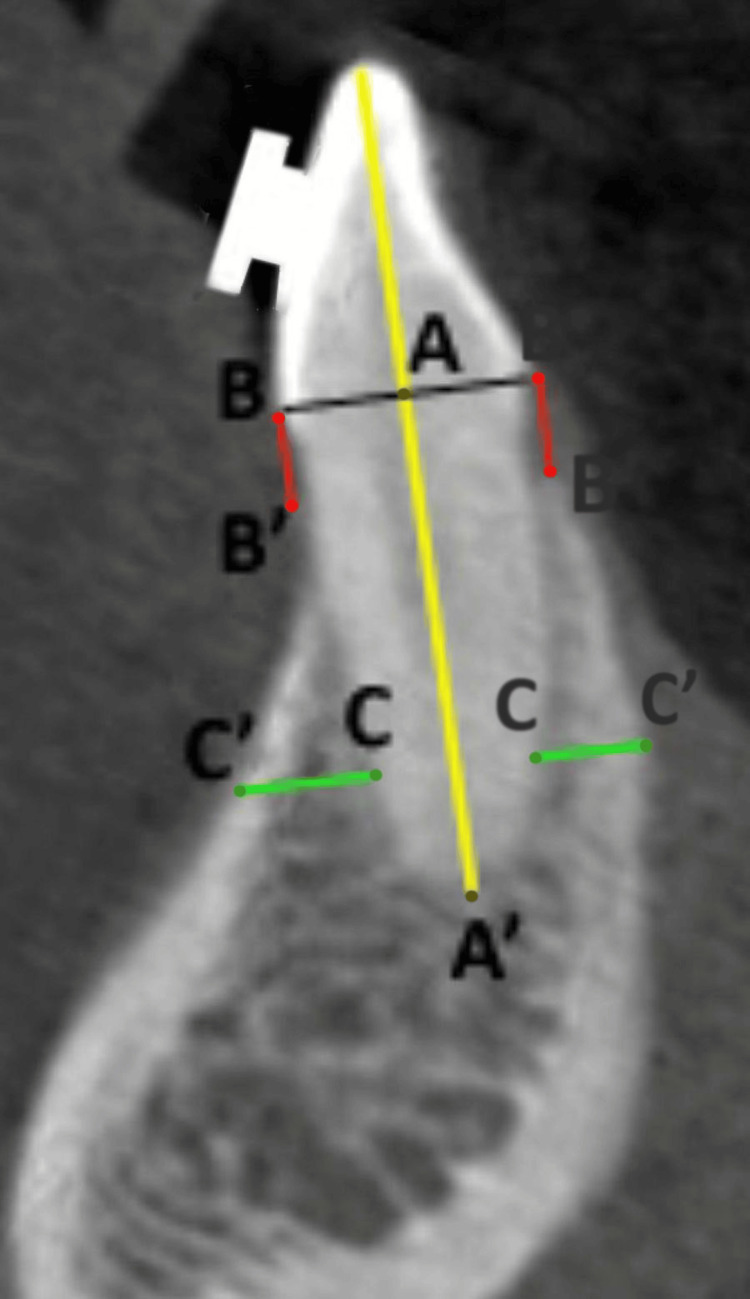
Cone-beam computed tomography image of mandibular central incisor showing measurement of root length (A-A') from the cementoenamel junction to the root apex along the long axis of the tooth, alveolar bone height (B-B') from the cementoenamel junction to the crest of the alveolar bone, and alveolar bone width (C-C') at 3 mm from the apex of the tooth on lingual and labial side. Measurements were done on both labial and lingual aspects. Original image from the case study.

Pre- and post-retraction CBCT scans were analyzed to measure the following three parameters: alveolar bone width, alveolar bone height, and root length of the mandibular incisors. Alveolar bone width was measured at the labial and lingual aspects of the incisors at 3 mm from the root apex. To assess alveolar bone width changes in the apical area of the incisors, alveolar bone width was measured at 3 mm from the root apex (C-C') on CBCT images. This distance was chosen because it lies within the apical third of the root, where bone remodeling and resorption are most relevant to periapical health and treatment outcomes. The 3-mm mark ensures sufficient bone volume for reliable measurement while avoiding artifacts near the apex. Alveolar bone height was assessed as the vertical distance from the cementoenamel junction (CEJ) to the alveolar crest on both the labial and lingual sides. The root length was measured from the CEJ to the root apex along the long axis of the tooth. All measurements were taken in the sagittal plane using multiplanar reconstruction (MPR) to ensure accuracy (Figure 1).

Reliability assessment

Intra- and inter-observer reliabilities were assessed to ensure the reliability of the measurements. Two trained orthodontists, blinded to patient details, independently performed the measurements on 10 randomly selected CBCT scans at two time points, one week apart. Intraclass correlation coefficients (ICCs) were calculated to evaluate measurement consistency. An ICC value of ≥0.80 was considered indicative of excellent reliability. Any discrepancies between observers were resolved by consensus or by averaging measurements. Calibration sessions were conducted before the study to standardize the measurement protocol and minimize variability.

Statistical analysis

The measured values were tabulated in Microsoft Excel (Microsoft Corporation, Redmond, WA, USA) and analyzed using the Statistical Package for the Social Sciences (SPSS) software Version 25 (IBM Corporation, Armonk, NY, USA). Descriptive statistics, including means, standard deviations, and ranges, were calculated for the alveolar bone width, height, and root length. The normality of continuous alveolar bone height and width measurements was assessed using the Kolmogorov-Smirnov test, with additional confirmation via Q-Q plots. Intergroup comparisons of the mean alveolar measurements across all four mandibular incisors (pre-retraction vs. post-retraction) were conducted using one-way analysis of variance (ANOVA). Paired t-tests were used for intragroup comparisons (pre- vs. post-retraction measurements of individual teeth). A significance threshold of p < 0.05 was applied for all analyses.

## Results

The study included 32 patients, 18 (56.2%) males and 14 (43.8%) females, with a mean age of 20.45 ± 5.20 years. Males were slightly older (21.75 ± 4.56 years) compared to females (19.56 ± 5.6 years), though this difference was not statistically tested. The sex distribution was balanced, with a moderate male predominance (Table 1). The age variability reflects a young adult population.

**Table 1:** Demographic characteristics of the study population. Categorical data are presented as frequency (n) and percentage (%). Continuous data are presented as mean and SD.

Parameters	Male	Female	Overall
Age (years), mean ± SD	21.75 ± 4.56	19.56 ± 5.60	20.45 ± 5.20
Sex, n (%)	18 (56.2%)	14 (43.8%)	32 (100%)

All parameters showed non-significant differences across the lower incisors (p > 0.05), both before and after retraction, indicating consistent bone and root characteristics between the incisors. However, the labial alveolar bone height post-retraction (p = 0.061) approached significance, suggesting a trend towards differential bone remodeling, although the difference was not statistically significant. A decrease in labial bone height (observed in all teeth) from the CEJ to the labial alveolar crest suggested apposition on the labial side, consistent with tooth movement towards the lingual side. The maximum apposition was observed in the left central incisor. An increase in alveolar bone height post-retraction, particularly on the lingual side, likely indicates lingual bone resorption due to lingual retraction forces, which increase the vertical distance from the CEJ to the lingual alveolar crest. The maximum resorption was observed in the left central incisor. For alveolar bone width, measured 3 mm from the root apex, minimal changes were observed post-retraction (p = 0.091 for labial, p = 0.464 for lingual), suggesting a stable bone width with no significant resorption or apposition at this level. Root length reduction post-retraction across all teeth (p = 0.224) indicated mild root resorption, a common consequence of orthodontic movement, but with no significant variation among teeth. The maximum root resorption was observed in the right and left central incisors (Table 2).

**Table 2:** Intragroup comparison of parameters between mandibular incisors using one-way ANOVA test. p > 0.05 denotes non-significance using one-way ANOVA test. Data are presented as mean ± SD. ANOVA, analysis of variance

Parameters	Time period of evaluation	Right central incisor	Right lateral incisor	Left central incisor	Left lateral incisor	F value	p-value
		Mean ± SD	Mean ± SD	Mean ± SD	Mean ± SD		
Labial alveolar bone height (mm)	Pre-retraction	2.17 ± 0.73	2.05 ± 0.37	2.13 ± 0.91	2.05 ± 0.59	0.24	0.861
	Post-retraction	1.72 ± 0.48	1.43 ± 0.60	1.08 ± 0.20	1.58 ± 0.38	1.52	0.061
Lingual alveolar bone height (mm)	Pre-retraction	2.48 ± 0.39	2.49 ± 0.25	2.45 ± 0.90	2.51 ± 0.18	0.95	0.417
	Post-retraction	2.87 ± 0.43	2.73 ± 0.34	2.97 ± 0.85	2.82 ± 0.57	0.07	0.975
Labial alveolar bone width (mm)	Pre-retraction	1.07 ± 0.38	0.97 ± 0.33	1.09 ± 0.41	0.96 ± 0.35	1.05	0.371
	Post-retraction	1.04 ± 0.81	0.61 ± 0.59	0.76 ± 0.74	0.68 ± 0.73	2.18	0.091
Lingual alveolar bone width (mm)	Pre-retraction	0.93 ± 0.27	1.23 ± 0.95	1.08 ± 1.04	1.14 ± 0.56	1.06	0.364
	Post-retraction	1.65 ± 0.81	1.76 ± 0.69	1.88 ± 0.93	1.53 ± 0.83	0.85	0.464
Root length (mm)	Pre-retraction	12.9 ± 2.18	13.48 ± 1.16	12.59 ± 2.19	13.49 ± 1.15	2.08	0.106
	Post-retraction	12.02 ± 1.12	12.58 ± 1.06	12.02 ± 1.30	12.47 ± 1.88	1.46	0.224

Intergroup comparisons using paired t-tests revealed statistically significant differences (p < 0.05) between the pre- and post-retraction measurements for most parameters in all teeth. The labial alveolar bone height was significantly reduced in all teeth (p < 0.05), demonstrating significant apposition of the bone on the labial side and reducing the distance between the CEJ and alveolar crest. The lingual alveolar bone height significantly increased in all mandibular incisors, indicating significant bone resorption at the crestal level. The labial alveolar bone width significantly decreased in all teeth (p < 0.05), suggesting movement of the root apex towards the labial side. The lingual alveolar bone width increased significantly in all the mandibular incisors. The root length was significantly decreased in all teeth. These findings indicate that orthodontic retraction of the mandibular incisors led to labial crestal bone apposition, lingual crestal bone resorption, and root resorption among the mandibular incisors (Table 3).

**Table 3:** Intergroup comparison of parameters for mandibular incisors using paired t-tests. *p < 0.05 denotes statistical significance using paired t-tests. Data are presented as mean ± SD.

Parameters	Time period of evaluation	Right central incisor	t-value	p-value	Right lateral incisor	t-value	p-value	Left central incisor	t-value	p-value	Left lateral incisor	t-value	p-value
		Mean ± SD			Mean ± SD			Mean ± SD			Mean ± SD		
Labial alveolar bone height (mm)	Pre-retraction	2.17 ± 0.73	2.91	0.005*	2.05 ± 0.37	4.97	0.001*	2.13 ± 0.91	6.37	0.001*	2.05 ± 0.59	3.78	0.003*
	Post-retraction	1.72 ± 0.48			1.43 ± 0.6			1.08 ± 0.2			1.58 ± 0.38		
Lingual alveolar bone height (mm)	Pre-retraction	2.48 ± 0.39	3.8	0.003*	2.49 ± 0.25	3.21	0.002*	2.45 ± 0.9	2.37	0.021*	2.51 ± 0.18	7.08	0.001*
	Post-retraction	2.87 ± 0.43			2.73 ± 0.34			2.97 ± 0.85			2.82 ± 0.57		
Labial alveolar bone width (mm)	Pre-retraction	1.07 ± 0.38	2.66	0.009*	0.97 ± 0.33	3.01	0.003*	1.09 ± 0.41	2.21	0.031*	0.96 ± 0.35	1.95	0.049*
	Post-retraction	1.04 ± 0.81			0.61 ± 0.59			0.76 ± 0.74			0.68 ± 0.73		
Lingual alveolar bone width (mm)	Pre-retraction	0.93 ± 0.27	4.77	0.001*	1.23 ± 0.95	2.55	0.013*	1.08 ± 1.04	3.24	0.001*	1.14 ± 0.56	2.2	0.031*
	Post-retraction	1.65 ± 0.81			1.76 ± 0.69			1.88 ± 0.93			1.53 ± 0.83		
Root length (mm)	Pre-retraction	12.9 ± 2.18	2.03	0.046*	13.48 ± 1.16	3.24	0.001*	12.59 ± 2.19	2.03	0.045*	13.49 ± 1.15	2.87	0.0005*
	Post-retraction	12.02 ± 1.12			12.58 ± 1.06			12.02 ± 1.3			12.47 ± 1.88		

## Discussion

The findings of this study indicated significant alterations in alveolar bone dimensions and root length following orthodontic retraction of the mandibular incisors, which is consistent with the literature on orthodontic tooth movement and its effects on periodontal structures [4,5]. However, these findings raise questions regarding the variability in bone response and the potential risks associated with retraction. The significant reduction in lingual alveolar bone height observed in this study is consistent with previous research on orthodontic retraction in bimaxillary protrusion. This could be because the retraction forces applied by TADs led to lingual tipping of the crown and the generation of tipping forces. Vardimon et al. [12] similarly reported a decrease in lingual crestal bone height during anterior maxillary tooth retraction, which was attributed to lingual movement of the crown due to tipping forces. The authors recommended using a 1:2 bone remodeling/tooth movement ratio as a guideline to determine the biocompatibility range of orthodontic tooth movement.

Retraction forces can lead to bone resorption, particularly in the crestal region, where the bone is the thinnest and most susceptible to remodeling. Bone resorption in orthodontic practice can introduce various potential treatment complications such as gingival recession, bone fenestration, dehiscence, and tooth mobility [10]. Because of the comparatively thin labial alveolar bone that encases the anterior segment, modifications to the anterior alveolar bone require particular consideration [13]. The present study revealed that the mean labial alveolar bone width ranged from 0.96 mm to 1.07 mm, whereas, for the lingual side, it ranged from 0.93 mm to 1.23 mm. Therefore, orthodontists should closely monitor changes in the alveolar bone after lower incisor retraction during orthodontic treatment, particularly in cases with a thin alveolar bone width during pre-retraction.

The use of maximum anchorage mechanics, such as TADs, in the current study likely intensified this effect by facilitating controlled en masse retraction, which concentrated the forces on the anterior segment. This is supported by Upadhyay et al. [14], who demonstrated that TAD-supported retraction enhances the efficiency of anterior tooth movement, but may exacerbate bone remodeling owing to the high forces involved. The consistency of these findings across studies underscores the biomechanical impact of retraction on the lingual crestal bone, particularly in patients with bimaxillary protrusion, in whom the initial protrusion of the incisors places the lingual bone under greater stress during treatment [1].

Conversely, the increase in labial alveolar bone height observed in most mandibular incisors in this study aligns with the concept of bone apposition on the tension side during orthodontic movement [15]. As the incisors were retracted, the lingual aspect of the alveolar bone experienced pressure forces, particularly at the crestal level, which could have stimulated bone resorption, as described by Melsen [6]. In contrast, the labial side experienced tensional forces, leading to bone apposition and an increase in alveolar bone levels [6]. Sun et al. [16] observed a notable reduction in alveolar bone height on the lingual aspect of mandibular incisors during retraction. However, the lack of significant intragroup differences suggests that all the lower incisors experienced similar effects under retraction forces. According to Garib et al. [17], the consequences of dental displacement on the alveolar bone, evaluated through the application of CBCT, delineate the boundaries of orthodontic practices and establish interventions that are permissible and contraindicated for each patient.

The significant increase in lingual alveolar bone width observed in this study is an intriguing finding that suggests movement of the root towards the labial cortical plate under the tipping forces exerted during retraction. The observed increase may be explained by adaptive remodeling in response to altered incisor position. This is consistent with controlled retraction mechanics, which aims to reposition the incisors within the existing alveolar envelope. This finding contrasts with that of Sarikaya et al. [4], who reported a non-significant change in alveolar bone width at apical area reduction following retraction. This discrepancy may be attributed to differences in methodology, as the current study focused on a relatively young and systemically healthy cohort with adequate bone volume. The evaluation was conducted using CBCT, which may have facilitated better evaluation than the 2D lateral cephalograms used in the study by Sarikaya et al. [4].

The decrease in labial alveolar bone width is another finding that requires further investigation. This reduction aligns with the biomechanical principle that the labial bone experiences compressive forces during retraction at the apical region, leading to a reduction in the labial alveolar bone width. In a study utilizing CBCT in patients receiving orthodontic interventions that included extraction of maxillary first premolars, Kuc et al. [18] reported a notable decrease in both the vertical and horizontal dimensions of the alveolar bone on the palatal aspect of the maxillary incisors after orthodontic treatment, which was associated with marked external root resorption. However, this contrasts with the study by Lund et al. [19], who reported minimal changes in lingual bone width, possibly because of less aggressive retraction protocols or differences in imaging modalities. The use of CBCT in the current study likely provided greater sensitivity in detecting these changes than 2D imaging used in earlier studies, which may explain the more pronounced findings.

A significant reduction in the root length across all mandibular incisors is a critical finding with important clinical implications. Root resorption is a well-documented risk factor for orthodontic treatment, particularly in cases involving significant tooth movements [10]. Motokawa et al. [20] posited that the interaction between the dental root and the cortical bone is crucial for instigating root resorption. This is consistent with the findings of Lu et al. [21], who reported significant root shortening in patients who underwent premolar extraction and retraction. Ballard et al. [22] demonstrated that the utilization of intermittent forces reduces the incidence of root resorption compared with the use of continuous forces. The application of nickel-titanium closed-coil springs, which deliver a consistent force, may also explain the root resorption observed in this study.

The lack of significant differences in intragroup comparisons across the mandibular incisors suggests a relatively uniform response to treatment, which supports the use of standardized retraction protocols. This is in line with the study by Eksriwong and Thongudomporn [23], who found consistent bone and root responses of 1:1 in the labial alveolar bone across the maxillary incisors in bimaxillary protrusion. These findings justify the use of CBCT for monitoring such changes, as it provides a comprehensive view of periodontal structures, overcoming the limitations of 2D imaging [8].

Clinical implications

The findings of this study have significant clinical implications for orthodontic treatment planning and execution in patients with class I bimaxillary protrusions undergoing first premolar extraction. The observed reductions in lingual alveolar bone height and root length highlight the need for careful monitoring during retraction to minimize iatrogenic complications such as root resorption and gingival recession. Clinicians should prioritize controlled retraction mechanics to optimize force distribution and limit excessive bone remodeling, particularly in patients with thin alveolar bone, such as those with skeletal class III malocclusion. The increase in labial bone height and lingual bone width in the apical area suggests that adaptive bone remodeling can support long-term periodontal stability, reinforcing the importance of positioning incisors within the alveolar housing ("with-the-bone" movement) to enhance periodontal health. The use of CBCT, as demonstrated in this study, is invaluable for precise pre- and post-treatment assessments, enabling orthodontists to tailor retraction strategies to individual anatomical variations and to mitigate risks. These insights underscore the necessity of integrating advanced imaging and biomechanical planning to achieve aesthetically pleasing and functionally stable outcomes, while minimizing adverse effects.

Limitations

However, this study had several limitations that warrant consideration. The inability to account for confounding variables, including discrepancies in patient adherence and mild variations in treatment methodologies, may have affected the results. The sample size of 32 patients, although statistically adequate, might not fully capture the diversity of anatomical and biological responses in a broader population, potentially limiting generalizability. The focus on a specific age group (18-30 years) excluded younger or older patients who may exhibit different bone remodeling patterns. Additionally, this study did not assess long-term stability or functional outcomes, such as changes in occlusion or periodontal health, beyond the treatment period, which are critical for evaluating the clinical success of retraction. Variations in growth patterns were not accounted for, and the omission of patients with skeletal class II and III malocclusions impeded the generalizability of the findings. Finally, although CBCT provides high-resolution data, radiation exposure remains a concern, and this study did not address the cost-effectiveness or accessibility of this imaging modality in routine practice.

## Conclusions

This study revealed significant morphometric changes in the mandibular incisors following retraction in patients with class I bimaxillary protrusions treated with first premolar extraction. The labial crestal bone showed significant apposition across all incisors, whereas the lingual crestal bone showed a reduction in all incisors, indicating bone remodeling. In contrast to the reduced labial bone width in all incisors, the lingual bone width increased significantly in the apical region, suggesting adaptive alveolar changes. The root length decreased significantly across all incisors, indicating the risk of resorption due to retraction forces. CBCT enables precise measurements that are indispensable for assessing these changes. These findings underscore the impact of controlled retraction with maximum anchorage on periodontal structures, highlighting the need for meticulous treatment planning to optimize aesthetic and functional outcomes while minimizing complications. Future research should investigate the long-term stability and methods to reduce the risk of resorption in these patients.
